# Postharvest Quality of *Citrus medica* L. (cv Liscia-Diamante) Fruit Stored at Different Temperatures: Volatile Profile and Antimicrobial Activity of Essential Oils

**DOI:** 10.3390/foods13111596

**Published:** 2024-05-21

**Authors:** Laura Quintieri, Michela Palumbo, Ilde Ricci, Bernardo Pace, Leonardo Caputo, Angelo Adduci, Anna Luparelli, Maria Cefola, Francesco Siano, Rosaria Cozzolino

**Affiliations:** 1 Institute of Sciences of Food Production, National Research Council of Italy (CNR), Via G. Amendola, 122/O, 70126 Bari, Italy; laura.quintieri@ispa.cnr.it (L.Q.); leonardo.caputo@ispa.cnr.it (L.C.); anna.luparelli@ispa.cnr.it (A.L.); 2 Institute of Sciences of Food Production, National Research Council of Italy (CNR), c/o CS-DAT, Via M. Protano, 71121 Foggia, Italy; michela.palumbo@ispa.cnr.it (M.P.); ilde.ricci@ispa.cnr.it (I.R.); bernardo.pace@ispa.cnr.it (B.P.); 3 Consorzio del Cedro di Calabria, Corso del Tirreno, 353, 87020 Santa Maria del Cedro, Italy; 4 Institute of Food Science, National Research Council of Italy (CNR), Via Roma 64, 83100 Avellino, Italy; rosaria.cozzolino@isa.cnr.it

**Keywords:** citron, essential oils, antimicrobials, terpenes, green peel color

## Abstract

Citron (*Citrus medica* L. cv. Liscia-diamante), cultivated in the “Riviera dei Cedri” (southern Italy), is mainly utilized in the production of candied fruit and essential oils (EOs). Up to now, no information regarding the effect of storage temperatures on citron has been reported. Here, citron samples, after harvesting, were stored at different temperatures (5, 10 and 20 °C at 70% relative humidity) for two weeks, and the main postharvest quality parameters were evaluated. Moreover, EOs extracted from the stored samples were chemically characterized to reveal changes in the volatiles profile and antimicrobial activity. The EOs presented monoterpene hydrocarbons (87.1 to 96.3% of the total oil profile) as the most abundant compounds, followed by oxygenated metabolites ranging from 9.7 to 3.1% of the total pattern. Postharvest quality traits showed a good retention of green peel color during storage at 5 °C, while EOs from samples stored for 7 and 14 days at 10 and 20 °C, respectively, showed the highest antimicrobial activity against most assayed strains. The results indicated storage at 10 °C for 7 days as the most suitable for the preservation of the postharvest quality of the fruit and the antimicrobial activity of the extracted EOs.

## 1. Introduction

*Citrus medica* L. (cv. Liscia-diamante), which recently received the European Protected Designation of Origin certification, is among the most popular citron varieties [1,2]. It is exclusively produced on a short stretch along the coast of high Tyrrhenian Sea, known as “Riviera dei Cedri” (Calabria, southern Italy). In this area, about the 98% of the national Italian production is concentrated, thanks to the moderate-warmth climate that has revealed the most suitable conditions for citron cultivation [2,3]. Citron is harvested at two different phenological phases: before maturity, when it has a green outer skin, and as a ripe fruit, with a yellow or yellow-green skin. Thus, two distinct productions are generated which have different intended uses. In general, the peel (flavedo) of green citron, which is rich in fragrance, is utilized in perfumery, pastry and culinary preparations, whereas the rind of mature citron is efficiently transformed in confectionary (candies and jams), drinks (cedrata), or salads [3,4]. Currently, the main problem faced by farmers is the preservation of fresh citron for as long as possible, avoiding any loss of its organoleptic characteristics, before using the green outer peel as a flavoring and coloring agent in pastry and in the preparation of culinary recipes [5].

Citron has been demonstrated to be highly susceptible to storage temperatures lower than 10–12 °C, even if cv. “Liscia-diamante” can withstand lower temperatures (about 8 °C) [6,7]. Although several postharvest technologies and low storage temperatures (8–14 °C) have been extensively applied to citrus fruits, such as lemons, tangerines and oranges, to reduce their metabolic activity, counteracting moisture loss and decays [8,9,10,11,12,13], no information regarding the effect of storage temperature on citron fruit has been described to date. Even if *Citrus medica* cv. “Liscia-diamante” is widely used both in the cosmetic sector and food industry, preservation procedures for fresh fruit to reduce waste and improve the quality of the final products are still lacking and represent a current challenge.

Citron is known worldwide for its peel fragrance that, like for other citrus species, is due to a unique mixture of volatile (mainly monoterpenes and sesquiterpenes) and non-volatile compounds which usually represent about 85–99% of the essential oil (EO) [4,14]. Limonene and γ-terpinene have been indicated as the main volatiles of the EO fraction extracted from the citron cv. “Liscia-diamante” by hydrodistillation (HD) and cold-pressing (CP), while citropten is the principal compound if the oil is obtained by supercritical carbon dioxide extraction (SFE) [4,15]. Chemical compounds identified in the peel extracts demonstrated several biological effects in in vitro and in vivo experiments [4,16,17]. EO from *C. medica* L. has been also reported as a remarkable inhibitor of pathogens and spoilers, suggesting that it can represent a valuable and low-cost natural antimicrobial agent which can be integrated in food products to guarantee microbial safety and quality [16,18,19,20]. Citrus oils, being a mixture of volatiles belonging to different chemical families, including unsaturated components, are generally unstable and their physical and chemical properties rely on different storage factors [20]. Experimental evidence accumulated over the years in cold-sensitive citrus species has shown that storage at low temperatures can cause substantial changes in the profiles of primary and secondary metabolites as part of the genetic and biochemical responses of fruit to handle low-temperature stress [21,22,23].

Specifically, variations in EO components during storage are affected by the volatility of metabolites, the plant species, the trichome structure and the specific storage conditions (temperature, oxygen availability and light) [20]. Generally, these changes have been ascribed to several reactions, including hydrolysis, oxidation, racemization, isomerization and rearrangement during storage [20].

It has been observed that some biologically active components of EOs can suddenly appear or completely disappear during storage, and for this, they have been indicated as chemical markers of the storage duration [24,25]. Starting from these findings, the aim of this study was to assess, for the first time, the effects of three different storage temperatures on the postharvest quality traits of citron fruit cv. “Liscia-diamante” and on the profile of volatile compounds of the EOs extracted from the stored fruit. Additionally, the antimicrobial activity of the EOs against some common pathogens was evaluated.

## 2. Methods

### 2.1. Samples and Storage Conditions

Citron fruits (*Citrus medica* L. cv. “Liscia-diamante”) collected from ten eight-year-old trees in Le Valline srls (Santa Maria del Cedro, Italy) were transported to the Postharvest Laboratory of CNR ISPA in Foggia to be processed. After discarding damaged citrons, 36 citron fruits were subdivided into three groups containing 12 citrons in each one, put in open plastic boxes and stored at 5, 10 and 20 °C, at 70% relative humidity for two weeks. Citron fruits were analyzed, as described below, immediately after harvest and during the storage (at 7 and 14 d) at each storage temperature.

### 2.2. Visual Quality and Color Attributes

Visual quality was evaluated on a 5-point rating scale, according to Cefola et al. [26], where 5: excellent (fresh appearance, full sensory acceptability); 4: good (product acceptable from a sensory point of view); 3: limit of sensory acceptability (limit of marketability); 2: product has notable visual defects; 1: severe visual defects.

For color attributes, an AP3200TPGE digital camera (JAI Ltd., Yokohama, Japan), placed inside a HPB60D photo studio box (HAVOX^®^, Vendôme, France), was used to capture images of fruit during storage (three replicates of three fruits for each measurement). The sensor of the camera was an RGB CMOS type, with a spatial resolution of 3.2 MP at 2 fps and a color depth of 24 bit/pixel. The focal length of the lens was 12 mm and F1.8 (KOWA Lens mod. LM12NC3 1/2), allowing a field of view (FOV) of 35 × 30 cm. Two LEDs, composed of 60 diodes in each one (HAVOX HPB60, 5500 K, 13,000 100 lumen CRI 93+), supplied the lighting. As a chromatic reference, a Color Checker (Passport Photo, 2 X-rite Italy srl, Prato Italy) with 24 known color stains was used in the camera’s field of view. The images captured by the digital camera were processed using Matlab^®^ R2021b (MathWorks Inc., Natick, MA, USA), following the method applied by Gonzalez et al. [27]. The raw image of each citron fruit was separated from the background and a binary image was generated using a specific algorithm that detaches the whole fruit, with a defined edge as the region of interest, and separates the three color components: red, green and blue (RGB). The data obtained in RGB were converted in the L* a* b* color scale: L* indicates the lightness from black (0 value) to white (100 value), a* the redness (+) or greenness (-) and b* the yellowness (+) or blueness (-). The Citron Color Index (CCI) was calculated using the formula proposed by Jiménez-Cuesta et al. [28]:

CCI = (1.000 × a*)/(L* × b*)

This parameter is used in the citrus industry to determine the harvesting date or to decide which fruit should undergo a degreening treatment [29].

### 2.3. Respiration Rate and Weight Loss

The respiration rate of citron stored at three different temperatures (5, 10 and 20 °C) was measured at harvest (0 d) and after 7 and 14 d of storage, as previously described by Kader [30]. In detail, the fruits, divided into replicates (*n* = 3) were placed into 3.6 L sealed plastic jars (one jar per replicate) where CO_2_ was allowed to accumulate up to 0.1 kPa (concentration of the CO_2_ standard). The analysis of CO_2_ was conducted by injecting 1 mL of gas sample from the headspace of the plastic jars into a gas chromatograph (p200 micro-GC-Agilent, Santa Clara, CA, USA) equipped with dual columns and a thermal conductivity detector. CO_2_ was analyzed with a retention time of 16 s and a total run time of 120 s on a 10 m porous polymer (PPU) column (Agilent, Santa Clara, CA, USA) at a constant temperature of 70 °C. The respiration rate was expressed as µmol CO_2_ kg^−1^ s^−1^.

The weight loss of each replicate was calculated as a percentage compared with weight at day 0.

### 2.4. Extraction and GC-FID Characterization of the Essential Oils from the Citron Fruit Peel (cv. “Liscia-Diamante”)

For each sample, the EOs were obtained by triturating, with a home blender, about 200 g of citron peel. Subsequently, 100 mL of distilled water was added. The water/essential oil emulsion obtained was centrifuged at 5000× *g* for 20 min, and the upper phase containing the EO was recovered and then dried by passage over sodium sulphate (Na_2_SO_4_).

EOs were analyzed by using a 7890A gas chromatographer (Agilent, Santa Clara, CA, USA) equipped with a flame ionization detector (FID) and split/splitless capillary inlet system. The apparatus was furnished with a 7683 Series autosampler system (Agilent, Santa Clara, CA, USA) containing a 10 µL syringe set at 1 µL for the delivery volume and at fast injection speed. Data acquisition was performed with GC ChemStation Rev. B.04.03-SP2 (Agilent) integration software. The compounds were separated using an RTX-5, 30 m × 0.25 mm × 0.25 μm column (Restek, Bellefonte, PA, USA). Then, 1 µL of EO diluted 1:50 (*v*/*v*) in *n*-hexane was injected in split mode 1:10. The carrier gas was helium at a constant flow of 1.0 mL min^−1^; nitrogen at 30 mL min^−1^ flow rate was the make-up gas. The oven temperature was initially set at 60 °C for 5 min, increased from 60 to 190 °C at 5 °C min^−1^ and kept for 5 min, then programmed from 190 to 290 °C at 15 °C min^−1^ and held for 15 min. The injector and detector temperatures were set at 250 and 280 °C, respectively. The identification of the volatile compounds was carried out using pure standards (where available) or by comparing the experimental retention times with data reported in the literature for the same compounds analyzed with the same instrumental conditions. The concentration was expressed in Area%.

### 2.5. Antimicrobial Activity of Essential Oils Extracted from Fresh and Stored Citron Fruit (cv. “Liscia-Diamante”)

EOs extracted from the peel of the fresh and stored citron were assayed to test the antimicrobial activity against 5 pathogens: *Yersinia enterocolitica subsp. enterocolitica* DSM4780, *Staphylococcus aureus* ATCC 6538P, *Listeria monocytogenes* LMG 23193 and *Candida albicans* DSM1386. *Escherichia coli* K12 was also included as a reference hygienic strain. Target strains used in the antimicrobial assays were obtained from the CNR-ISPA Microbial Collection (http://www.ispacnr.it/en/microbial-collection/; accessed on 1 June 2023) and stored at −80 °C. Before their use, all strains were freshly cultured overnight under aerobic conditions as follows: Luria Bertani (LB) broth (Biolife Italiana, Milan, Italy) at 37 °C, 150 strokes min^−1^ for bacteria and Potato Dextrose Agar (PDA) (Biolife Italiana, Milan, Italy) at 25 °C for *Candida albicans*.

The strains were screened using the agar disk diffusion assay method according to EUCAST guidelines [31] and following the method described by Mitropoulou et al. [16] with slight modifications. Briefly, a sterile cotton swab soaked with 0.5 McFarland solution of each strain was spread on Petri dishes with Muller Hinton agar (tryptone, 17.5 g L^−1^; beef extract, 2 g L^−1^; soluble starch, 1.5 g L^−1^ and agar, 17 g L^−1^). Then, cellulose discs (Oxoid, Cambridge, UK) were soaked with 20 µL of undiluted essential oils from different samples. Citral (C10H16O, CAS number: 5392-40-5) (≥96%, Food Chemicals Codex, FCC, natural, food grade, FG; Sigma-Aldrich (Milan, Italy) and the related 500-, 100- and 10-fold dilution in methanol were also evaluated. Plates were incubated at 37 °C for 16–20 h. Diameters (nearest millimeter) of inhibition zones around the assayed disks were measured with a caliper from the back of the plate held above a dark background. Alternatively, diameters of inhibition halos were digitally measured on the high-resolution images of the inoculated plates by using University of Texas Health Science Center at San Antonio (UTHSCSA) ImageTool 3.0 software [31].

### 2.6. Statistical Data Analysis

Two-way ANOVA for *p* ≤ 0.05 was performed to evaluate the effects of temperature (5, 10 and 20 °C), storage time (7 and 14 d) and their interaction on the quality parameters and on the volatile composition of the EOs extracted from the citron peel. When the interaction between factors was significant, data were shown as graphs with mean values ± standard deviation. Regarding the EO data, when the interaction was significant, the combined effect of storage (0, 7 and 14 d) and temperature (5, 10 and 20 °C) was evaluated by performing a one-way ANOVA for *p* ≤ 0.05. The mean values (*n* = 3) were separated using the least significant difference (LSD) test (*p* ≤ 0.05), and Statgraphics Centurion (version 18.1.12, Warrenton, VA, USA) was used for statistical analyses.

## 3. Results and Discussion

### 3.1. Postharvest Quality Parameters of Citrons Fruit (cv. “Liscia-Diamante”) Stored at Three Different Temperatures

The results of the two-way ANOVA performed on the postharvest quality parameters of citron fruit stored at three different temperatures (5, 10 and 20 °C) showed that the storage time (S) and the temperature (T) had the main effects (*p* ≤ 0.0001) both on the respiration rate and on the Citron Color Index (CCI; Table 1). Moreover, as highlighted in Table 1, the interaction (T × S) significantly affected both of the attributes.

Visual quality (only influenced by the storage time, Table 1) showed a reduction in all storage conditions. Specifically, the mean value of the visual quality was higher than the limit of marketability (score 3) at the end of the storage period in all samples, regardless of the storage temperature.

**Figure 1:**
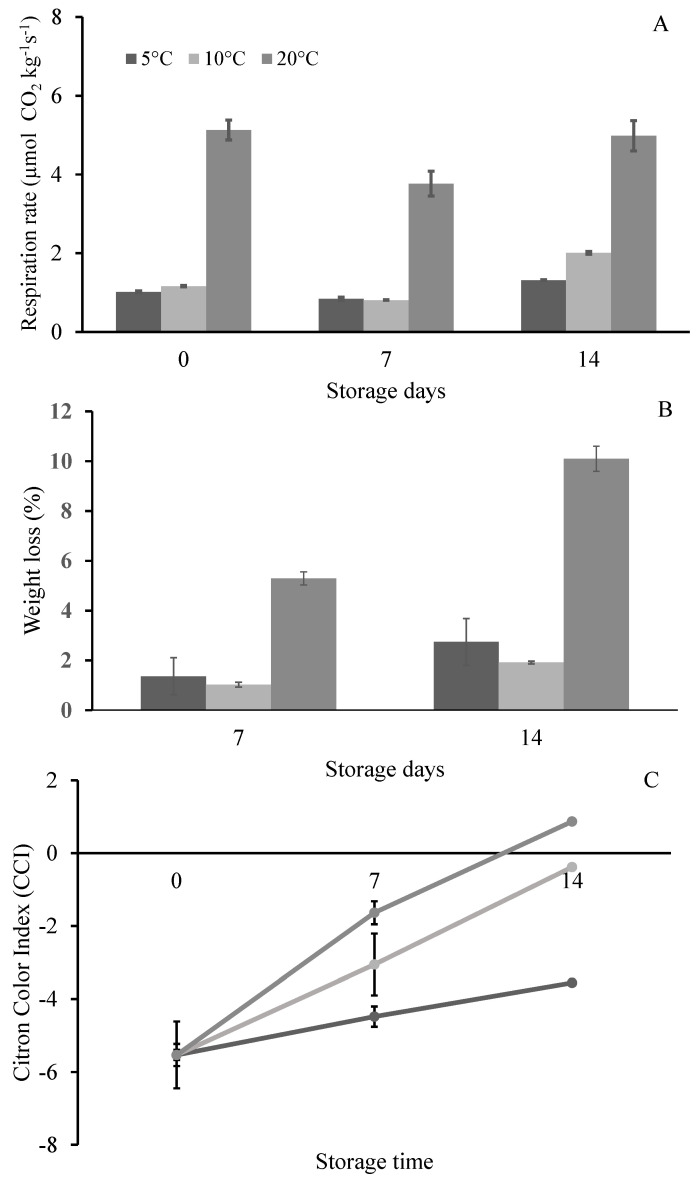
Respiration rate (**A**), weight loss (**B**) and Citron Color Index, CCI (**C**) of citron cv. “Liscia-diamante” stored for 7 and 14 d at 5, 10 and 20 °C. Data are expressed as the mean value of three replicates ± standard deviation.

By comparing the different conditions, the respiration rate of the fruit preserved at 20 °C showed an initial mean value of about 5.13 ± 0.25 µmol CO_2_ kg^−1^ s^−1^, which was higher with respect to the samples stored at 5 and 10 °C, for which values of 1.02 ± 0.02 and 1.16 ± 0.02 µmol CO_2_ kg^−1^ s^−1^, respectively, were observed (Figure 1A). The respiration rate remained almost stable after 7 d, with a slight increase after 14 d of storage at each temperature, confirming the non-climacteric nature of citron fruit, in line with other citrus species (Figure 1A) [11]. On the other hand, changes in the peel color occurred during storage, as previously reported for lemon fruit [32].

Storage at low temperature (5 or 10 °C) preserved the weight loss, which showed a significant increase in fruit stored at 20 °C, reaching the values of about 6 and 10% after 7 and 14 days, respectively (Figure 1B).

During storage, an increase in CCI was generally measured at each temperature. Moreover, significant differences among samples at each storage time (7 and 14 d) were observed (Figure 1C). For fruit stored at 10 and 20 °C, the CCI values increased by about 2.5-fold from 7 to 14 d, even though, at the end of the storage, only the samples at 20 °C showed a positive value of CCI (Figure 1C). Similar results were reported on lemon by Mitalo et al. [33], who showed that the color of the fruit peel gradually changed, from green to yellow, during the storage at different temperatures (5–20 °C), together with an increase in the CCI value. The authors highlighted that at 15 °C, the peel degreening was more evident, followed by the samples stored at 10 and 20 °C; on the contrary, at 5 °C, the lemon peel retained the green color even after 6 weeks of storage.

**Figure 2:**
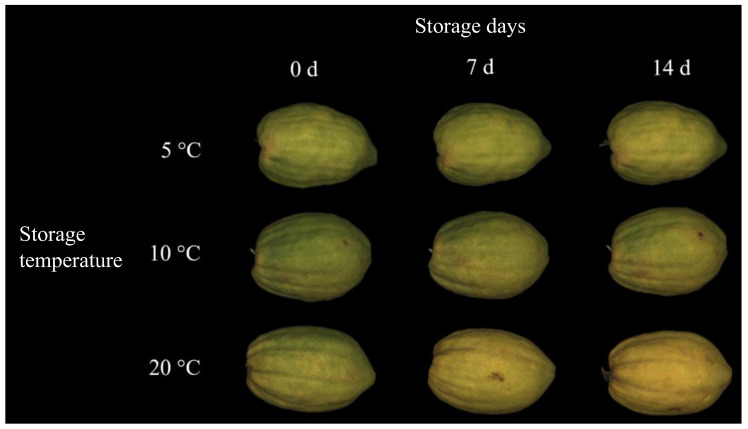
Citron fruits cv. “Liscia-diamante” at harvest (0 d) and during storage (7 and 14 d) at different temperatures (5, 10 and 20 °C).

Color data were also confirmed by the visual appearance of the fruit. Citron stored at 20 °C showed a more yellow skin with respect to the samples preserved at 5 and 10 °C (Figure 2). As previously described for other citrus fruit, color changes in the peels observed during the postharvest storage might have been caused by possible endogenous ethylene production [34]. Starting from these very preliminary results and in agreement with earlier data on other citrus species, the “pseudoclimacteric” behavior of the citron cv. “Liscia-diamante” might be speculated. As, to the best of our knowledge, this is the first study reporting the postharvest performance of citron fruit cv. “Liscia-diamante”, further experiments should be performed to assess the putative “pseudoclimacteric” behavior of this cultivar.

Finally, as for other citrus fruit, citron might be picked at internal full maturation, at the immature stage, or with a greenish rind and ripened during storage until (degreening process) a desirable color is obtained for the users [11].

### 3.2. Changes in the Profile of Essential Oils in Fresh and Stored Citron Fruit (cv. “Liscia-Diamante”)

**Figure 3:**
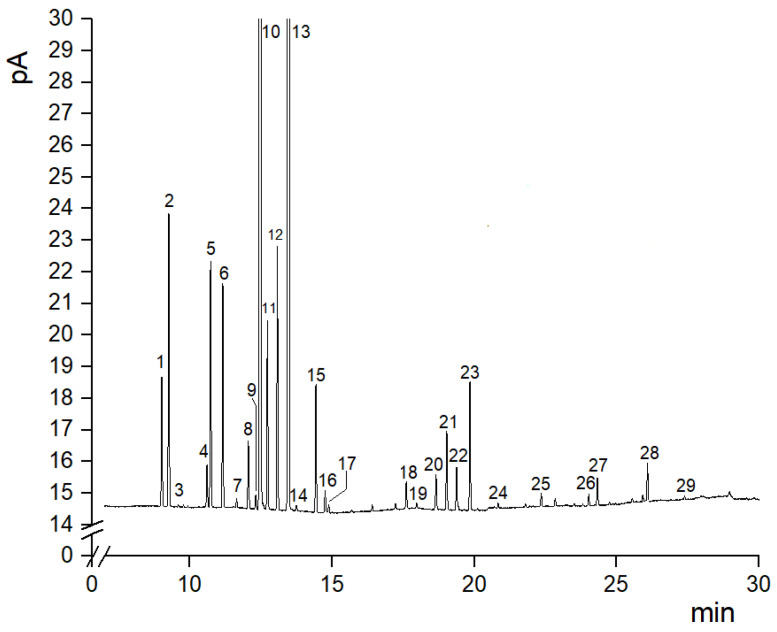
Typical gas chromatographic profile of the essential oil extracted from citron cv. “Liscia-diamante”. Numbers on the peaks refer to Table 2.

The qualitative and semi-quantitative profiles of the volatile content of each EO sample extracted from the citron peel was investigated by GC-FID. Figure 3 displays a typical GC profile of the EOs extracted from the citron.

Twenty-nine volatile components were identified in the chemical fraction for each sample, as listed in Table 2 in order of elution. Data, expressed as the relative percentage of the peak areas (% Area), without considering the non-volatile residue, demonstrated that the detected compounds constituted nearly 98% of the whole oil profile in each sample (Table 2).

In general, the EO profiles obtained from the citron stored at different times and temperatures showed the same main components, but to a different extent (Table 2). In all EO samples, the most abundant volatile compounds were monoterpene hydrocarbons (MHs), which ranged from 87.1 to 96.3%. The oxygenated metabolites (OMs) represented about 9.7–3.1%, with monoterpene aldehydes being predominant among these components (1.7–7.8%), followed by monoterpene alcohols (0.4–1.5%) (Table 2). The term “terpene aldehydes” mainly denotes neral and geranial, the so-called “citral” constituents that define the sensory peculiarity of lemon oil [3]. Esters, represented by citronellyl, neryl and geranyl acetate, ranged from about 0.4 to 0.8%, while aliphatic aldehydes (nonanal and undecanal) showed contents of about 0.05–0.2%. Sesquiterpene hydrocarbons (SHs) (β-bisabolene and germacrene B) were the least abundant, accounting for about 0.3–0.5%. Both the SHs were previously detected in *C. medica* L. cv. “Liscia-diamante” with a very low abundance, in agreement with our results [4]. In each sample, limonene was the principal compound, with the lower percentage in the fresh fruit (48%) and the higher amount in the sample stored at 10 °C for 7 d (64%). The second most abundant volatile in all samples was γ-terpinene, which ranged from 20 to 28% (Table 2). These findings are in line with earlier results which indicated a chemotype limonene/γ-terpinene for the EO extracted from the peel of *C. medica* L. cv. “Liscia-diamante” [3,4]. α-Tujene (0.91–1.05%), α-pinene (2.21–2.45%), β-pinene (1.87–2.11%), myrcene (1.30–1.81%), *cis*-β-ocimene (1.19–1.77%), *trans*-β-ocimene (1.62–2.48%) and terpinolene (0.89–1.04%) were the other main MHs (Table 2).

To evaluate the effect of the temperature (T), the storage time (S) and their interaction (T × S), a two-way ANOVA was performed on the GC-FID semi-quantitative data. Results showing the components individually affected by each factor (T and S) and by their interaction (T × S) are reported in Supplementary Table S1. For the compounds influenced by the interactions of the two factors (T × S), a one-way ANOVA (Table 3) was carried out in comparison with fresh fruit.

By contrast, for the compounds which did not show significant changes regarding the interaction (T × S), the effect of each factor was considered (Table 4).

The results reported in Table 3 highlighted significant differences among the amount of some volatile compounds contained in the EOs from fresh fruit compared to EOs from samples stored at different temperatures (5, 10 and 20 °C) at 7 and 14 d. Specifically, related to the fresh samples, EOs obtained from the stored fruit showed significantly higher values for several MHs, including myrcene, limonene, *cis*- and *trans*-β-ocimene, which, excepted for limonene, did not show significant variations with respect to the different storage temperatures and storage times (Table 3). On the other hand, EOs from stored samples showed decreased values of γ-terpinene, nonanal, neral, geranial, citronellyl acetate and germacrene B compared to EOs from fresh fruits. Again, no statistical difference was found among the concentration of these metabolites along the storage, regardless of either temperature or storage duration (Table 3). The trend showed by both MH and OM metabolites agrees with previous data which reported that during the ripening stage of different citrus species, the chemical composition of the EOs undergoes to some changes which generally consist of an increase in MHs and a decrease in MOs [35,36,37,38]. Recently, Ghani et al. [39] has showed that the content of limonene, which presented the highest value in the EOs of the green citron, decreased in relation to the maturity stage and even in over-ripe citron. Although citron is classified as a non-climacteric fruit, as it stops the maturation process at harvest, data shown in Table 2 suggest the presence of possible auto-induced ethylene production in the peel of the stored citrons. These results are consistent with those reported for the color changes in the peel observed along the preservation time (Figure 1B) and seem to suggest the possible “pseudoclimacteric” behavior of the citron cv. “Liscia-diamante”. As reported for other citrus fruit, ethylene, in fact, is described to play a role in regulating the ripening of citrus peel by stimulating the respiration, the breakdown of chlorophyll and the accumulation of carotenoids [34].

Table 4, which lists the metabolites affected by each factor (T or S), shows that the temperature had a significant influence on several volatile components. Specifically, β-myrcene (grassy and metallic aroma), neryl acetate (floral) and undecanal (aldehydic) showed an average content statistically higher in the EOs extracted from peels of samples stored at 5 °C, while *trans*-sabinene hydrate (woody, balsamic), linalool (floral, fruity and sweet aroma), neral and geranial (both with a lemon aroma) were found at higher amounts in fruit preserved at 20 °C (*p* ≤ 0.001, *p* ≤ 0.05), with the main effect on the two monoterpene aldehydes (*p* ≤ 0.0001) (Table 4).

Regarding the influence of the storage time on the chemical composition of the EOs, from 7 to 14 d, a statistical increase in the average amount of sabinene (woody, herbaceous and spicy), β-pinene (green, smoky and woody aroma), neral, geranial and undecanal was detected, whereas a decrease in the concentration of β-myrcene, *trans*-sabinene hydrate, linalool, α-terpineol (citrus flavor) and geraniol (floral) was measured (Table 4). The different trends shown by several constituents belonging to the same chemical class seem to suggest that a “pseudoclimateric” process is still active in citron peel, regardless of the storage conditions. In detail, Wu et al. [35] revealed that the chemical composition of EOs from the peel of *C. medica* L. (cv. *sarcodactylis*) differed significantly in three different ripening periods. At the fully mature stage, the content of MH increased, but the amount of total sesquiterpenes and total MOs decreased. Moreover, Marzocchi et al. [38], studying the effect of the maturation on the chemical composition of the EOs from two different Italian cultivars of bergamot (*Citrus × Bergamia*), observed an increase with harvesting time for two MOs (named nerol and citronellyl acetate), which, however, statistically decreased in the first stage of ripening. The behavior of these oxygenated components can be considered in agreement with the data reported in Table 4 for the same compounds, as they were recorded only along two weeks. Overall, it is important to highlight that to confirm the possible “pseudoclimateric” behavior of citron cv. “Liscia-diamante”, it is necessary to take up further investigations aimed to verify putative endogenous ethylene production during fruit conservation.

### 3.3. Antimicrobial Activity of Essential Oils against Pathogens

EOs obtained from the peels of citron immediately after harvest and from fruit stored at 5, 10 and 20 °C for 7 and 14 d, respectively, were assayed for their antimicrobial activity against five strains of human pathogens. EOs registered an inhibitory activity against four out of five assayed strains; no activity was registered against *Y. enterocolitica* DSM 4780 (Table 5), in line with other studies on EOs from the peel [40] and the aerial parts [41] of *C. medica* L.

EOs obtained from samples stored at 5 °C were not active at 7 d, although their constituents were significantly increased with respect to the fresh fruit. The antimicrobial activities of EOs from citron stored at 4 °C were only registered against *L. monocytogenes* and *E. coli* at 14 d of storage. In contrast, EOs obtained from samples stored at 20 °C for 14 d showed halo diameters higher than 1 cm against all sensitive strains. These findings are in agreement with the chemical analysis reported in Table 3, which shows that limonene amounts significantly increased in comparison with those detected in the EOs from fresh fruit, although they were almost stable regardless of the time and temperature applied. Recently, Guo et al. [42,43] have also reported strong anti-listerial activity extended by EOs extracted from fingered citron, which was shown to mainly contain D-limonene and other terpenes. Specifically, the authors showed that microscopy analyses highlighted the lysis and the collapse of the bacterial membrane following treatment with EOs (4%, *v*/*v*) from citron. On the other hand, transcriptome analyses revealed that adaptive responses of *L. monocytogens*, involving flagellum assembly, motility, the alteration of carbohydrates and metallic cation uptake, were induced by the same concentration of EOs from fingered citron, which was also shown to destroy the intact architecture of biofilm and reduce the biofilm biomass, the thickness and the substratum coverage [44].

EOs and the relative main ingredients, including d-limonene, β-pinene and γ-terpinene, of *Citrus medica* × *limonum* inhibited *E. coli*, increasing bacterial cell membrane permeability and surface hydrophobicity and causing intracellular protein and nucleic acid leakage [45,46]. Likewise, limonene showed potent inhibitory activity in *S. aureus,* impairing the bacterial respiratory metabolism and inhibiting ATP synthesis [47]. Furthermore, the potential of limonene as an inhibitor of efflux pump involved in antibiotic resistance by *S. aureus* was also demonstrated [47].

The anticandidal activity exhibited by EOs from citrus was previously demonstrated at concentrations between 125 and 500 µg mL^−1^. Interestingly, this effect was directly related to limonene’s ability to damage the *C. albicans* cell wall/membrane and modify cellular adhesion and plasticity [48].

Furthermore, EOs from the peel of the citron samples stored at 10 °C for 7 d inhibited the growth of *L. monocytogenes, C. albicans* and *E. coli* and the multidrug-resistant (MDR) *S. aureus* target strain. After 14 d, EOs from samples stored at 10 °C registered inhibitory activity only against *S. aureus* and *L. monocytogenes.* The chemical analysis of EOs revealed the highest concentration of limonene (64%) in the fruit stored at 10 °C for 7 d (Table 5), suggesting that this MH is responsible for the antimicrobial activity, consistent with some evidence about the antimicrobial activity of limonene against *L. monocytogenes*, *C. albicans* and *S. aureus* that has already been reported [49,50,51,52]. These data can support the use of EOs from citron as an ingredient in novel formulations to counteract the antibiotic resistance spread, enhance the antifungal efficacy of pharmaceutical formulates and ultimately to increase agricultural produce shelf life in the postharvest period.

## 4. Conclusions

This work represents a first preliminary report on the postharvest performance of citron cv. “Liscia-diamante”, in which the visual quality, the volatile compound profile and the microbial activity of EOs in relation to different times and temperatures of storage were evaluated. Based on the obtained results, storage at 5 °C seemed to preserve the green color of the citron peel, while higher temperatures (10 and 20 °C) appeared to promote yellowing, probably due to the putative production of endogenous ethylene. Data on the respiration rate confirmed the non-climacteric nature of citron fruits, in line with other citrus species. In any case, according to the changes in the color of the citron peel and consistent with the variation in the profiles of the volatile components detected in the EOs during storage, the “pseudoclimacteric” behavior of citron cv. “Liscia-diamante” might be speculated. Since citron stored at 10 or 20 °C cannot be used for industrial transformation (candying, in particular), it could represent a valuable source of EOs with biological properties. The results indicated that conservation at 10 °C for 7 days could be considered the optimal storage parameter to preserve the postharvest quality of the fruit and the antimicrobial activity of the extracted EOs. Specifically, the antimicrobial efficacy of the EOs from *Citrus medica* L. cv. “Liscia-diamante”, observed here against some common pathogens, including multidrug-resistant species, can suggest that the EOs from this citron can be promising as ingredients for the development of a pharmaceutical formulation aimed to counteract antibiotic resistance in clinical settings, but also as a component used to increase the shelf life of horticultural products in the postharvest period.

## Figures and Tables

**Table 1:** Effect of temperature (T), storage time (S) and their interaction (T × S) on the quality attributes of citron fruit (cv. “Liscia-diamante”).

Quality Attributes	Temperature (T)	Storage Time (S)	Interaction (T × S)
Visual quality	ns	***	ns
Respiration rate (µmol CO_2_ kg^−1^ s^−1^)	****	****	***
Citron Color Index (CCI)	****	****	*

Within each row, each factor and their interaction are significantly different for *p* ≤ 0.05 (*), *p* ≤ 0.001 (***), *p* ≤ 0.0001 (****), ns = not significant.

**Table 2:** Volatile compounds and their relative peak area (% A) in the essential oils obtained from citron fruit (cv. “Liscia-diamante”) at harvest (0 d) and during storage (7 and 14 d) at different temperatures (5, 10 and 20 °C).

Volatile Compounds	N. Peak	0 d			7 d									14 d								
		Fresh			5 °C			10 °C			20 °C			5 °C			10 °C			20 °C		
α-Tujene	1	1.04	±	0.03	1.03	±	0.01	0.91	±	0.01	0.91	±	0.01	1.03	±	0.11	1.00	±	0.08	1.05	±	0.01
α-Pinene	2	2.21	±	0.04	2.45	±	0.03	2.16	±	0.09	2.14	±	0.06	2.43	±	0.23	2.32	±	0.08	2.40	±	0.01
Camphene	3	0.02	±	0.00	0.02	±	0.00	0.02	±	0.00	0.02	±	0.00	0.02	±	0.00	0.02	±	0.00	0.02	±	0.00
Sabinene	4	0.19	±	0.22	0.35	±	0.01	0.35	±	0.04	0.34	±	0.01	0.37	±	0.02	0.38	±	0.06	0.37	±	0.02
β-Pinene	5	2.11	±	0.01	2.02	±	0.08	1.86	±	0.14	1.87	±	0.04	2.04	±	0.16	2.05	±	0.25	2.08	±	0.07
Myrcene	6	1.30	±	0.01	1.81	±	0.05	1.80	±	0.02	1.69	±	0.03	1.73	±	0.06	1.66	±	0.14	1.69	±	0.00
α-Phellandrene	7	0.06	±	0.00	0.08	±	0.01	0.06	±	0.01	0.07	±	0.01	0.07	±	0.00	0.08	±	0.01	0.08	±	0.01
α-Terpinene	8	0.59	±	0.01	0.53	±	0.05	0.47	±	0.02	0.48	±	0.00	0.52	±	0.03	0.51	±	0.04	0.55	±	0.01
*p*-Cimene	9	0.10	±	0.03	0.10	±	0.04	0.07	±	0.01	0.07	±	0.00	0.09	±	0.04	0.11	±	0.02	0.08	±	0.01
Limonene	10	47.60	±	0.05	60.79	±	2.79	63.58	±	1.21	61.65	±	0.43	59.59	±	0.11	61.90	±	0.13	57.28	±	0.96
*cis*-β-Ocimene	11	1.19	±	0.04	1.50	±	0.01	1.49	±	0.01	1.46	±	0.03	1.73	±	0.13	1.44	±	0.04	1.77	±	0.15
*trans*-β-Ocimene	12	1.62	±	0.04	2.11	±	0.01	2.14	±	0.02	2.05	±	0.04	2.43	±	0.18	2.05	±	0.05	2.48	±	0.23
γ-Terpinene	13	28.03	±	0.29	22.47	±	2.10	19.99	±	0.99	20.06	±	0.10	22.09	±	1.14	21.29	±	0.01	22.99	±	0.64
*trans*-Sabinene hydrate	14	0.05	±	0.00	0.05	±	0.02	0.06	±	0.02	0.07	±	0.01	0.03	±	0.00	0.05	±	0.00	0.06	±	0.01
Terpinolen	15	1.04	±	0.01	1.00	±	0.09	0.91	±	0.05	0.89	±	0.01	0.98	±	0.04	0.95	±	0.01	1.02	±	0.03
Linalool	16	0.26	±	0.01	0.17	±	0.06	0.21	±	0.05	0.28	±	0.06	0.13	±	0.04	0.18	±	0.03	0.20	±	0.00
Nonanal	17	0.13	±	0.00	0.06	±	0.02	0.03	±	0.01	0.06	±	0.00	0.08	±	0.01	0.06	±	0.01	0.06	±	0.00
Citronellal	18	0.25	±	0.01	0.18	±	0.07	0.23	±	0.07	0.32	±	0.06	0.12	±	0.03	0.16	±	0.08	0.12	±	0.01
Terpinen-4-ol	19	0.03	±	0.00	0.04	±	0.01	0.03	±	0.01	0.05	±	0.01	0.09	±	0.03	0.04	±	0.00	0.05	±	0.00
α-Terpineol	20	0.24	±	0.29	0.22	±	0.11	0.51	±	0.49	0.54	±	0.09	0.13	±	0.06	0.13	±	0.06	0.23	±	0.00
Neral	21	2.72	±	0.01	0.63	±	0.02	0.57	±	0.30	1.21	±	0.08	0.98	±	0.39	0.93	±	0.01	1.35	±	0.04
Geraniol	22	0.47	±	0.08	0.27	±	0.16	0.59	±	0.62	0.65	±	0.18	0.13	±	0.07	0.08	±	0.04	0.27	±	0.04
Geranial	23	4.80	±	0.01	1.03	±	0.02	0.93	±	0.50	2.03	±	0.10	1.69	±	0.69	1.49	±	0.01	2.30	±	0.04
Undecanal	24	0.05	±	0.05	0.03	±	0.01	0.02	±	0.00	0.03	±	0.00	0.05	±	0.01	0.03	±	0.01	0.04	±	0.01
Citronellyl acetate	25	0.44	±	0.10	0.10	±	0.01	0.14	±	0.06	0.14	±	0.01	0.27	±	0.04	0.22	±	0.05	0.21	±	0.04
Neryl acetate	26	0.11	±	0.01	0.11	±	0.01	0.09	±	0.03	0.07	±	0.00	0.10	±	0.03	0.10	±	0.03	0.06	±	0.00
Geranyl acetate	27	0.26	±	0.01	0.25	±	0.01	0.25	±	0.03	0.23	±	0.01	0.25	±	0.04	0.20	±	0.08	0.21	±	0.02
β-Bisabolene	28	0.43	±	0.04	0.32	±	0.01	0.33	±	0.04	0.32	±	0.03	0.36	±	0.04	0.27	±	0.12	0.30	±	0.03
Germacrene B	29	0.10	±	0.01	0.03	±	0.01	0.03	±	0.01	0.03	±	0.00	0.04	±	0.01	0.03	±	0.00	0.02	±	0.00

Data are mean values (*n* = 3) ± standard deviation.

**Table 3:** Significant differences in the content (relative peak area, % A) of the volatile compounds identified in the essential oils from citron fruit (cv. “Liscia-diamante”) at harvest (0 d) and during storage (7 and 14 d) at different temperatures (5, 10 and 20 °C).

	0 d		7 d								14 d				
Volatile Compounds	Fresh		5 °C		10 °C		20 °C		5 °C		10 °C		20 °C		*p*
Myrcene	1.30	b	1.81	a	1.80	a	1.69	a	1.73	a	1.66	a	1.69	a	**
γ-Terpinene	0.59	a	0.53	abc	0.47	bc	0.48	c	0.52	bc	0.51	c	0.55	ab	*
Limonene	47.60	d	60.79	abc	63.58	a	61.65	ab	59.59	bc	61.90	ab	57.28	c	***
*cis*-β-Ocimene	1.19	c	1.50	b	1.49	b	1.46	b	1.73	a	1.44	b	1.77	a	**
*trans*-β-Ocimene	1.62	c	2.11	b	2.14	b	2.05	b	2.43	a	2.05	b	2.48	c	**
A-Terpinolene	28.03	a	22.47	b	19.99	c	20.06	c	22.09	bc	21.29	bc	22.99	b	***
Nonanal	0.13	a	0.06	b	0.03	c	0.06	b	0.08	b	0.06	b	0.06	b	***
Terpinen-4-ol	0.03	b	0.04	b	0.03	b	0.05	b	0.09	a	0.04	b	0.05	b	*
Neral	2.72	a	0.63	c	0.57	c	1.21	b	0.98	bc	0.93	bc	1.35	b	****
Geranial	4.80	a	1.03	d	0.93	d	2.03	bc	1.69	bcd	1.49	cd	2.30	b	****
Citronellyl acetate	0.44	a	0.10	c	0.14	c	0.14	c	0.27	b	0.22	bc	0.21	bc	**
Germacrene B	0.10	a	0.03	b	0.03	b	0.03	b	0.04	b	0.03	b	0.02	b	****

For each compound, the mean values followed by different letters (a, b, c, d) are significantly different (*p* ≤ 0.05) according to the least significant difference (LSD) test. Significance: * *p* ≤ 0.05, ** *p* ≤ 0.01, *** *p* ≤ 0.001, **** *p* ≤ 0.0001.

**Table 4:** Effect of temperature (5, 10 and 20 °C) or storage time (7 and 14 d) on the content (relative peak area, % A) of the volatile compounds in the essential oils extracted from the citron fruit (cv. “Liscia-diamante”).

Volatile Compounds	Temperature							Storage Time				
	5 °C		10 °C		20 °C		*p*	7 d		14 d		*p*
Sabinene	0.36	ns	0.36	ns	0.35	ns	ns	0.34	b	0.37	a	*
β-Pinene	2.03	ns	1.95	ns	1.98	ns	ns	1.92	b	2.05	a	*
Myrcene	1.77	a	1.73	ab	1.69	b	*	1.76	a	1.69	b	**
*trans*-Sabinene hydrate	0.04	b	0.05	a	0.06	a	**	0.06	a	0.05	b	*
Linalool	0.15	c	0.19	b	0.24	a	***	0.22	a	0.17	b	**
α-Terpineol	0.18	b	0.32	ab	0.38	a	ns	0.42	a	0.16	b	**
Neral	0.80	b	0.75	b	1.28	a	****	0.80	b	1.08	a	***
Geraniol	0.20	b	0.33	ab	0.46	a	ns	0.50	a	0.16	b	**
Geranial	1.36	b	1.21	b	2.16	a	****	1.33	b	1.82	a	***
Undecanal	0.04	a	0.02	b	0.03	a	**	0.03	b	0.04	a	**
Neryl acetate	0.10	a	0.10	a	0.07	b	**	0.09	ns	0.09	ns	ns

For each volatile compound, the mean values followed by different letters (a, b, c) are significantly different (*p* ≤ 0.05) according to the least significant difference (LSD) test. Significance: * *p* ≤ 0.05, ** *p* ≤ 0.01, *** *p* ≤ 0.001, **** *p* ≤ 0.0001, ns = not significant.

**Table 5:** Antimicrobial activity of essential oils extracted from fresh and stored citron fruit (cv. “Liscia-diamante”) during storage (7 and 14 d) at different temperatures (5, 10 and 20 °C).

Strains	Halos (mm) *						
	0 d	7 d			14 d		
	Fresh	5 °C	10 °C	20 °C	5 °C	10 °C	20 °C
*Yersinia enterocolitica subsp. enterocolitica* DSM4780	n.d **	n.d.	n.d	n.d.	n.d.	n.d.	n.d.
*Staphylococcus aureus* ATCC 6538P	n.d.	n.d.	9 ± 1	n.d.	n.d.	9 ± 1	11 ± 2
*Listeria monocytogenes* LMG 23193	n.d.	n.d.	7 ± 1	n.d.	10 ± 2	7 ± 2	11 ± 1
*Candida albicans* DSM1386	n.d.	n.d.	8 ± 2	n.d.	n.d.	n.d.	12 ± 2
*Escherichia coli* K12	n.d.	n.d.	8 ± 2	n.d.	7 ± 2	n.d.	11 ± 1

* The values represent means ± standard deviation (*N* = 3) of inhibition halos calculated by subtracting the disc surface area. ** n.d. = not detected.

## Data Availability

The original contributions presented in the study are included in the article, further inquiries can be directed to the corresponding author.

## Supplementary Materials

The following supporting information can be downloaded at: https://www.mdpi.com/article/10.3390/foods13111596/s1.

## Abbreviations

PDO	Protected Designation of Origin
EOs	Essential oils
MHs	Monoterpene hydrocarbons
CCI	Citron Color Index
GC-FID	Gas Chromatography–Flame Ionization Detector
ANOVA	Analysis of variance
MDR	Multidrug resistant

## References

[1] Reg. Ue 2023/971, 10/05/2023—COMMISSION IMPLEMENTING REGULATION (EU) 2023/971 of 10 May 2023 Entering a Name in the Register of Protected Designations of Origin and Protected Geographical Indications (‘Cedro di Santa Maria del Cedro’ (PDO)). Official Journal of the European Union on the 10/05/2023. http://eur-lex.europa.eu/legal-content/EN/TXT/HTML/?uri=OJ:L:2023:132:TOC.

[2] Continella A., Goldschmidt E.E., Bar-Joseph M. (2023). The Citron in Italy and Its Cultivation in Calabria. The Citron Compendium: The Citron (Etrog) Citrus medica L.: Science and Tradition.

[3] Gabriele B., Fazio A., Dugo P., Costa R., Mondello L. (2009). Essential oil composition of *Citrus medica* L. cv. Diamante (Diamante citron) determined after using different extraction methods. J. Sep. Sci..

[4] Gullo G., Tribulato E., Inglese P. (2012). Ricerca: Cedro. Gli agrumi: Botanica, Storia e arte, Alimentazione, Paesaggio, Coltivazione, Ricerca, Utilizzazione, Mondo e Mercato.

[5] Brigand J.P., Nahon P. (2016). Gastronomy and the citron tree (*Citrus medica* L.). Int. J. Gastron. Food Sci..

[6] Klein J.D., Hebbe Y., Shapovalov A., Shklyar G., Korol L., Cohen S. (2012). Changes in peel color of citron fruits from different genetic origins in response to postharvest copper and gibberellic acid treatments. VII International Postharvest Symposium, Kuala Lumpur (Malaysia).

[7] Tundis R., Xiao J., Silva A.S., Carreiró F., Loizzo M.R. (2023). Health-Promoting Properties and Potential Application in the Food Industry of *Citrus medica* L. and Citrus × clementina Hort. Ex Tan. Essential Oils and Their Main Constituents. Plants.

[8] Klein J.D., Yaniv Z., Dudai N. (2014). Citron Cultivation, Production and Uses in the Mediterranean Region. Medicinal and Aromatic Plants of the Middle-East. Medicinal and Aromatic Plants of the World.

[9] Zacarias L., Cronje P.J., Palou L., Talon M., Caruso M., Gmitter F.G. (2020). Postharvest technology of citrus fruits. The Genus Citrus.

[10] D’Aquino S., Palma A., Agabbio M., Tijskens L.M.M., Vollebregt H.M. (2003). Response of Three Citrus Species to Different Hygrometric Conditions. Proceedings of the International Conference on Quality in Chains. An Integrated View on Fruit and Vegetable Quality.

[11] Ladaniya M., Ladaniya M. (2010). Preharvest Factors Affecting Fruit Quality and Postharvest Life. Citrus Fruit: Biology, Technology, and Evaluation.

[12] El-Otmani M., Ait-Oubahou A., Zacarías L., Yahia E.M. (2011). Citrus spp.: Orange, mandarin, tangerine, clementine, grapefruit, pomelo, lemon, and lime. Postharvest Biology and Technology of Tropical and Subtropical Fruits.

[13] Patel B., Tandel Y.N., Patel A.H., Patel B.L. (2016). Chilling injury in tropical and subtropical fruits: A cold storage problem and its remedies: A review. Int. J. Sci. Environ..

[14] González-Mas M.C., Rambla J.L., López-Gresa M.P., Blázquez M.A., Granell A. (2019). Volatile Compounds in Citrus Essential Oils: A Comprehensive Review. Front. Plant Sci..

[15] Menichini F., Tundis R., Bonesi M., de Cindio B., Loizzo M.R., Conforti F., Statti G.A., Menabeni R., Bettini R., Menichini F. (2011). Chemical composition and bioactivity of *Citrus medica* L. cv. Diamante essential oil obtained by hydrodistillation, cold-pressing and supercritical carbon dioxide extraction. Nat. Prod. Res..

[16] Mitropoulou G., Fitsiou E., Spyridopoulou K., Tiptiri-Kourpeti A., Bardouki H., Vamvakias M., Panas P., Chlichlia K., Pappa A., Kourkoutas Y. (2017). *Citrus medica* essential oil exhibits significant antimicrobial and antiproliferative activity. LWT.

[17] Caputo L., Quintieri L., Cavalluzzi M.M., Lentini G., Habtemariam S. (2018). Antimicrobial and antibiofilm activities of citrus water-extracts obtained by microwave-assisted and conventional methods. Biomedicines.

[18] Mitropoulou G., Nikolaou A., Santarmaki V., Sgouros G., Kourkoutas Y. (2020). *Citrus medica* and *Cinnamomum zeylanicum* essential oils as potential biopreservatives against spoilage in low alcohol wine products. Foods.

[19] Wu K., Jin R., Bao X., Yu G., Yi F. (2021). Potential roles of essential oils from the flower, fruit and leaf of *Citrus medica* L. var. sarcodactylis in preventing spoilage of Chinese steamed bread. Food Biosci..

[20] Jangi F., Ebadi M.T., Ayyari M. (2021). Qualitative changes in hyssop (*Hyssopus officinalis* L.) as affected by cold plasma, packaging method and storage duration. J. Appl. Res. Med. Aromat. Plants.

[21] Obenland D.M., Collin S.H., Sievert J., Fjeld K., Doctor J., Arpaia M.L. (2008). Commercial packing and storage of navel oranges alters aroma volatiles and reduces flavour quality. Postharvest Biol. Technol..

[22] Obenland D., Collin S., Sievert J., Arpaia M.L. (2013). Mandarin flavor and aroma volatile composition are strongly influenced by holding temperature. Postharvest Biol. Technol..

[23] Lado J., Gurrea A., Zacarías L., Rodrigo M.J. (2019). Influence of the storage temperature on volatile emission, carotenoid content and chilling injury development in Star Ruby red grapefruit. Food Chem..

[24] Rosado L.D.S., Pinto J.E.B.P., Bertolucci S.K.V., Jesus H.C.R.D., Alves P.B. (2013). Changes in the content and composition of the essential oil of *Ocimum basilicum* L. during storage. J. Essent. Oil Res..

[25] Ebadi M.T., Sefidkon F., Azizi M., Ahmadi N. (2017). Packaging methods and storage duration affect essential oil content and composition of lemon verbena (*Lippia citriodora* Kunth.). Food Sci. Nutr..

[26] Cefola M., Carbone V., Minasi P., Pace B. (2016). Phenolic profiles and postharvest quality changes of fresh-cut radicchio (*Cichorium intybus* L.): Nutrient value in fresh vs. stored leaves. J. Food Comp. Anal..

[27] Gonzalez R.C., Woods R.E., Eddins S.L. (2004). Digital Image Processing Using MATLAB. https://www.cin.ufpe.br/~sbm/DEN/Digital%20Image%20Processing%20Using%20Matlab%20(Gonzalez).pdf.

[28] Jiménez-Cuesta M., Cuquerella J., Martinez-Javaga J.M., Matsumoto K. (1982). Determination of a color index for citrus fruit degreening. Proceedings of the International Society of Citriculture. International Citrus Congress.

[29] DOGV, Diari Oficial de la Comunitat Valenciana. **2006**, *5346*, 30321–30328. http://hdl.handle.net/10251/38426.

[30] Kader A.A., Kader A.A. (2002). Methods of gas mixing, sampling and analysis. Postharvest Technology of Horticultural Crops.

[31] Cavalluzzi M.M., Budriesi R., De Salvia M.A., Quintieri L., Piarulli M., Milani G., Gualdani R., Micucci C.I., Rosato A., Viale M. (2021). Lubeluzole: From anti-ischemic drug to preclinical antidiarrheal studies. Pharmacol. Rep..

[32] Nasrin T.A.A., Arfin M.S., Rahman M.A., Molla M.M., Sabuz A.A., Matin M.A. (2023). Influence of novel coconut oil and beeswax edible coating and MAP on postharvest shelf life and quality attributes of lemon at low temperature. Meas. Food.

[33] Mitalo O.W., Otsuki T., Okada R., Obitsu S., Masuda K., Hojo Y., Matsuura T., Mori I.C., Abe D., Asiche W.O. (2020). Low temperature modulates natural peel degreening in lemon fruit independently of endogenous ethylene. J. Exp. Bot..

[34] Paul V., Pandey R., Srivastava G.C. (2012). The fading distinctions between classical patterns of ripening in climacteric and non-climacteric fruit and the ubiquity of ethylene—An overview. J. Food Sci. Technol..

[35] Wu Z., Li H., Yang Y., Zhan Y., Tu D. (2013). Variation in the components and antioxidant activity of *Citrus medica* L. var. *sarcodactylis* essential oils at different stages of maturity. Ind. Crops Prod..

[36] Venturini N., Barboni T., Curk F., Costa J., Paolini J. (2014). Volatile and flavonoid composition of the peel of *Citrus medica* L. var. Corsican fruit for quality assessment of its liqueur. Food Technol. Biotechnol..

[37] Di Rauso Simeone G., Di Matteo A., Rao M.A., Di Vaio C. (2020). Variations of peel essential oils during fruit ripening in four lemon (*Citrus limon* (L.) Burm. F.) cultivars. J. Sci. Food Agric..

[38] Marzocchi S., Baldi E., Crucitti M.C., Toselli M., Caboni M.F. (2019). Effect of harvesting time on volatile compounds composition of bergamot (*Citrus× Bergamia*) essential oil. Flavour Fragr. J..

[39] Ghani A., Taghvaeefard N., Hosseinifarahi M., Dakhlaoui S., Msaada K. (2022). Essential oil composition and antioxidant activity of citron fruit (*Citrus medica* var. macrocarpa Risso.) peel as relation to ripening stages. Int. J. Environ. Health Res..

[40] Fratianni F., Cozzolino A., De Feo V., Coppola R., Ombra M.N., Nazzaro F. (2019). Polyphenols, antioxidant, antibacterial, and biofilm inhibitory activities of peel and pulp of *Citrus medica* L., *Citrus bergamia*, and *Citrus medica* cv. Salò cultivated in southern Italy. Molecules.

[41] Al-Mariri A., Safi M. (2014). In vitro antibacterial activity of several plant extracts and oils against some gram-negative bacteria. Iran. J. Med. Sci..

[42] Guo J.J., Gao Z.P., Xia J.L., Ritenour M.A., Li G.Y., Shan Y. (2018). Comparative analysis of chemical composition, antimicrobial and antioxidant activity of citrus essential oils from the main cultivated varieties in China. LWT.

[43] Guo J., Hu X., Gao Z., Li G., Fu F., Shang X., Liang Z., Shan Y. (2021). Global transcriptomic response of *Listeria monocytogenes* exposed to Fingered Citron (*Citrus medica* L. var. *sarcodactylis Swingle*) essential oil. Food Res. Int..

[44] Gao Z., Zhong W., Chen K., Tang P., Guo J. (2020). Chemical composition and anti-biofilm activity of essential oil from *Citrus medica* L. var. *sarcodactylis Swingle* against *Listeria monocytogenes*. Ind. Crops Prod..

[45] Tang W., Zhang Z., Nie D., Liu S., Li Y., Liu M., Zhang Y., Ou N., Li Y. (2023). Selective antibacterial activity of Citrus Medica limonum essential oil against Escherichia coli K99 and Lactobacillus acidophilus and its antibacterial mechanism. LWT.

[46] Ambrosio C.M.S., Contreras-Castillo C.J., Da Gloria E.M. (2020). In vitro mechanism of antibacterial action of a citrus essential oil on an enterotoxigenic Escherichia coli and Lactobacillus rhamnosus. J. Appl. Microbiol..

[47] Han Y., Chen W., Sun Z. (2021). Antimicrobial activity and mechanism of limonene against Staphylococcus aureus. J. Food Safety.

[48] Pedroso R.D.S., Balbino B.L., Andrade G., Dias M.C.P.S., Alvarenga T.A., Pedroso R.C.N., Pimenta L.P., Lucarini R., Pauletti P.M., Januário A.H. (2019). In vitro and in vivo anti-*Candida* spp. activity of plant-derived products. Plants.

[49] Leite M.C.A., Bezerra A.P.D.B., Sousa J.P.D., Guerra F.Q.S., Lima E.D.O. (2014). Evaluation of antifungal activity and mechanism of action of citral against Candida albicans. Evid. Based Complement. Altern. Med..

[50] OuYang Q., Tao N., Zhang M. (2018). A damaged oxidative phosphorylation mechanism is involved in the antifungal activity of citral against *Penicillium digitatum*. Front. Microbiol..

[51] Thakre A., Zore G., Kodgire S., Kazi R., Mulange S., Patil R., Shelar A., Santhakumari B., Kulkarni M., Kharat K. (2018). Limonene inhibits *Candida albicans* growth by inducing apoptosis. Med. Mycol..

[52] de Araújo A.C.J., Freitas P.R., Barbosa C.R.d.S., Muniz D.F., Ribeiro-Filho J.J., Tintino S.R., Júnior J.P.S., Filho J.M.B., de Sousa G.R., Coutinho H.D.M. (2021). Modulation of Drug Resistance by Limonene: Inhibition of Efflux Pumps in *Staphylococcus aureus* Strains RN-4220 and IS-58. Curr. Drug Metab..

