# Supplementary material for: Postharvest Quality of Citrus medica L. (cv Liscia-Diamante) Fruit Stored at Different Temperatures: Volatile Profile and Antimicrobial Activity of Essential Oils
Source: Foods. 2024 May 21;13(11):1596. doi: 10.3390/foods13111596 (PMC11171597; doi:10.3390/foods13111596)
Supplement: Supplementary file 1 [file foods-13-01596-s001.zip › foods-3001117-SI.pdf]

Supplementary material

Table S1. - Effect of temperature (T, 5, 10 and 20 °C), storage time (S, 7 and 14 d), and their interaction (T x S) on the quality attributes and on the volatile compounds detected in the essential oils (EOs) extracted from citron cv Liscia- diamante .

**Quality attributes**		**Temperature (T)**	**Storage time (S)**	**Interaction (T x S)**
Visual quality		ns	***	ns
Respiration rate (μmol CO_2 kg^{-1} s^{-1})		****	****	***
Citron Color Index (CCI)		****	****	*
**Volatile compounds**	**N.peak**			
α-Tujene	1	*	**	*
α-Pinene	2	**	**	*
Camphene	3	ns	ns	ns
Sabinene	4	ns	*	ns
β-Pinene	5	ns	*	ns
Myrcene	6	*	**	ns
α-Phellandrene	7	ns	**	**
α-Terpinene	8	*	**	*
p-Cimene	9	ns	ns	ns
Limonene	10	****	****	*
cis-β-Ocimene	11	***	****	***
trans-β-Ocimene	12	**	****	***
γ-Terpinene	13	*	**	**
trans-Sabinene hydrate	14	**	*	ns
Terpinolen	15	**	***	**
Linalool	16	***	**	ns
Nonanal	17	***	**	*
Citronellal	18	*	****	*
Terpinen-4-ol	19	**	***	**
α-Terpineol	20	ns	**	ns
Neral	21	****	***	ns
Geraniol	22	ns	**	ns
Geranial	23	****	***	ns
Undecanal	24	**	**	ns
Citronellyl acetate	25	ns	****	*
Neryl acetate	26	**	ns	ns
Geranyl acetate	27	ns	ns	ns
β-Bisabolene	28	ns	ns	ns
Germacrene B	29	*	ns	**

Within each row, each factor and their interaction are significantly different for *$p \leq 0.05$, **$p \leq 0.01$, ***$p \leq 0.001$, ****$p \leq 0.0001$, ns = not significant.
